# Molecular Level Understanding of Amine Structural Variations on Diaminodiphenyl Sulfone to Thermomechanical Characteristics in Bifunctional Epoxy Resin: Molecular Dynamics Simulation Approach

**DOI:** 10.3390/polym17121694

**Published:** 2025-06-18

**Authors:** Hei Je Jeong, Sung Hyun Kwon, Jihoon Lim, Woong Kwon, Gun Hwan Park, Eunhye Lee, Jong Sung Won, Man Young Lee, Euigyung Jeong, Seung Geol Lee

**Affiliations:** 1 Department of Materials Science and Engineering, Ulsan National Institute of Science and Technology (UNIST), Ulsan 44919, Republic of Korea; 2 School of Chemical Engineering, Pusan National University, Busan 46241, Republic of Korea; sunghyun.kwon@pusan.ac.kr (S.H.K.);; 3 Department of Organic Material Science and Engineering, Pusan National University, Busan 46241, Republic of Korea; 4 Department of Textile System Engineering, Kyungpook National University, Daegu 41566, Republic of Korea; 5 Defense Material and Energy Development Center, Agency for Defense Development, Yuseong, P.O. Box 35, Daejeon 34060, Republic of Korea

**Keywords:** epoxy resin, molecular dynamics (MD), diglycidyl ether of bisphenol F (DGEBF), diaminodiphenyle sulfone (DDS), thermomechanical properties

## Abstract

Epoxy-based composite materials, widely used in various industries such as coatings, adhesives, aerospace, electronics, and biomedical engineering, remain a topic of global interest due to their varying characteristics based on the base resin and curing agents used. This paper employs molecular dynamics simulation to examine the thermal and mechanical properties, as well as molecular behaviors, of epoxy systems cured with diglycidyl ether of bisphenol F as the base resin and aromatic amine curing agents, specifically the meta structure of 3,3′-diaminodiphenyl sulfone (3,3′-DDS) and the para structure of 4,4′-diaminodiphenyl sulfone (4,4′-DDS). The 3,3′-DDS system demonstrated a greater density and Young’s modulus than the 4,4′-DDS system. This tendency was analyzed based on differences in molecular fractional free volume and cohesive energy density (CED). The 4,4′-DDS system exhibits a higher glass transition temperature (T_g_) compared to the 3,3′-DDS system, with values of 406.36 K and 431.22 K, respectively. To understand this behavior, we examined atomic-scale displacements at T_g_ through mean squared displacement analysis, which revealed that the onset of molecular motion occurs at a lower temperature in the 3,3′-DDS system. Molecular-level study reveals how the structural features of each curing agent appear in thermal and mechanical properties, offering important insights for epoxy system development.

## 1. Introduction

Epoxy resins, known for their versatility as thermosetting materials, have gained global attention for their wide-ranging applications across industries, including coatings, adhesives, aerospace, electronic materials, and biomedical applications [1,2,3,4]. Additionally, epoxy-based materials play a pivotal role in modern aircraft, particularly in the aerospace industry, where their unique properties contribute to the development of high-performance components. Epoxy resins, known for their exceptional adhesive and mechanical characteristics, are widely utilized in the construction of critical structural elements in both commercial and military aircraft [1,5,6]. Epoxy resins are often employed as matrices in composite materials, where they form a strong bond with reinforcing fibers such as carbon or glass [5,6,7]. This synergy creates lightweight yet robust composite structures. The mechanical properties of epoxy composites, including high tensile strength and stiffness, make them well-suited for applications where both durability and weight efficiency are vital.

The ultimate properties of epoxy resin are from the specific three-dimensional network structure formed while the epoxy system performs crosslinking reaction between base epoxy resin and curing agents [8,9,10]. The type of the base resin varies depending on the number of epoxy rings present in the molecule, categorized as bi-, tri-, or tetra-functional corresponding to 2, 3, or 4 epoxide rings, respectively [11]. The properties of the base resin change with the number of epoxide rings, influencing its functionality [11]. Among the many kinds of epoxy resin, the bifunctional epoxy system is relatively straightforward and easy to handle; hence it is one of the most used base epoxy resins in industry. It can also be crosslinked by various curing agents, including aromatic amines, acid anhydrides, phenols, cycloaliphatic amines, aliphatic amines, and thiols [12,13,14,15]. The most common aromatic amine is diaminediphenyl sulfone (DDS), which exists in two stereoisomeric forms: 3,3′-diaminediphenyl sulfone (3,3′-DDS) and 4,4′-diaminediphenyl sulfone (4,4′-DDS). These isomers share the same chemical formula but differ in their configurational structures, known as meta and para configurations, respectively. Despite having the same molecular weight, epoxy systems crosslinked with these isomers exhibit different performances [16,17,18,19,20].

To elucidate these differences, this study employs molecular dynamics simulations to quantitatively analyze the intrinsic molecular behavior of epoxy networks. In particular, we examine how subtle differences in cross-linking positions, specifically between the meta and para configurations which differ only in their bonding positions, affect molecular mobility and interactions. This variation in molecular behavior leads to significant differences in thermomechanical properties, an aspect that previous studies have not clearly elucidated. Achieving this understanding requires experimentation under diverse conditions. However, high costs, time constraints, and significant variability in characteristics based on process conditions make it challenging to conduct comprehensive tests [21,22].

Molecular dynamics (MD) simulations have emerged as a versatile computational tool with applications spanning materials science, drug discovery, flexible electronics, and energy storage research [23,24,25,26]. While traditionally employed in these diverse fields for predicting molecular interactions and material behaviors, the rigorous application of MD modeling to epoxy resins provides unique insights into structure–property relationships [27,28,29,30,31]. For example, Fan et al. used MD simulation to investigate the thermomechanical characteristics of a bifunctional base resin and Jeffamin-230 curing agent epoxy system at various conversion levels and temperatures [32]. Similarly, Li et al. predicted and experimentally verified the thermal and mechanical properties of an epoxy system composed of a bifunctional base resin and various curing agents [28,33]. Kwon et al. used MD simulation to investigate the thermomechanical characteristics and diffusion behavior of an epoxy system to determine the impact of various curing agents [34]. Additionally, Odegard’s study analyzed three unique epoxy systems, comparing their properties to experimental data to validate the predictions made by MD modeling simulation [5,29]. As demonstrated by these studies, MD simulations are a valuable tool for predicting material properties based on multiple variables in a timely and cost-effective manner prior to undertaking direct testing. Analyzing behavior at the molecular level provides insights into the principles that underpin material properties. Several research groups have shown that simulations can reliably predict parameters, notably for epoxy systems, with good agreement between simulation and experimental results [5,31,35].

In this study, we focus on understanding the characteristics of epoxy systems based on the degree of curing, employing a relatively simple bifunctional epoxy, DGEBF, and aromatic amines, 3,3′-DDS and 4,4′-DDS. To construct epoxy polymer models, MD simulation techniques were utilized in conjunction with the PCFF force field. These models underwent validation, and properties were determined using conditions similar to experimental setups. The gathered properties were analyzed to unveil trends in epoxy systems with increasing degrees of curing, as well as behaviors and variations in attributes induced by the curing agent. The molecular-level investigation aimed to uncover differences in behavior based on the structure of the curing agents and to comprehend the fundamental principles driving each characteristic.

## 2. Computational Details

### 2.1. Model Preparations

**Figure 1:**
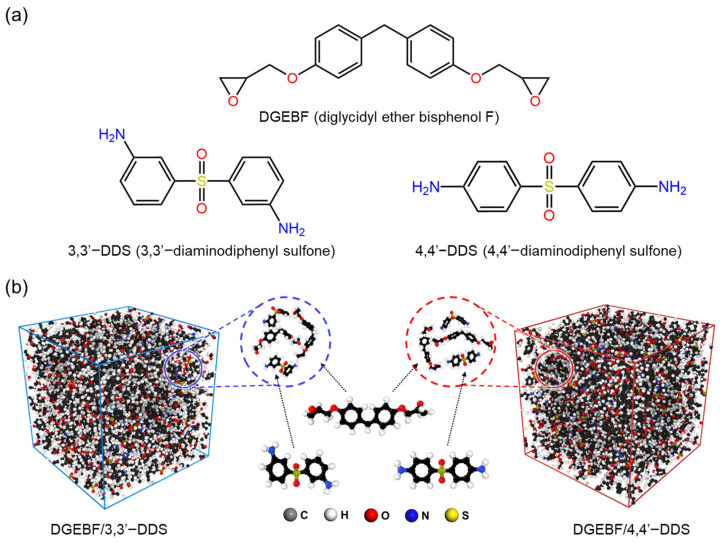
(**a**) Molecular structures of base resin (DGEBF) and curing agents (3,3′-DDS and 4,4′-DDS) and (**b**) optimized and equilibrated simulation model of DGEBF/3,3′-DDS and DGEBF/4,4′-DDS.

All the MD simulation epoxy system’s initial models were created on a full-atomistic scale. Figure 1a shows chemical structures of base resin and curing agents composing the epoxy system of this research, which are DGEBF, 3,3′-DDS and 4,4′-DDS. The initial epoxy system model was composed of certain amounts of base resin molecules and the curing agent molecules. Figure 1b demonstrates the initial amorphous models of each curing agent with DGEBF base resin constructed by Amorphous Cell module in Materials Studio [36], which is a Monte Carlo method with periodic boundary conditions. The number of base resin and curing agent molecules in each amorphous cell are stoichiometrically composed, 200 and 100, respectively.

### 2.2. Simulation Details

The PCFF was applied to describe the epoxy resin and curing agents, as PCFF is a well-fitted force field for describing epoxy system [37,38,39,40]. The total energy (E_total_) of the epoxy system’s molecular structure, consisting of several energy terms, was calculated by Equation (1):

(1) $$E_{total}=E_{vdW}+E_{Q}+E_{bond}+E_{torsion}+E_{inversion}+E_{bond-bond}+E_{angle-bond}+E_{angle-angle}+E_{angle-torsion}+E_{bond-torsion}$$

where $E_{vdW},\;E_{Q},\;E_{bond},\;E_{torsion},\;E_{inversion},\;E_{bond-bond},\;E_{angle-bond},\;E_{angle-angle},\;E_{angle-torsion,\;}$ and $E_{bond-torsion}$ are van der Waals, electrostatic, bond-stretching, angle-bending, torsion, out of plane bending angle, cross term between two bonds, coupling between bond and angle, two angles with a common bond, cross term for angle and torsion, and the bond and torsion angle, respectively. MD calculations were conducted using the following: the Large-scale Atomic/molecular Massively Parallel Simulator (LAMMPS) [41] code, developed by S. Plimpton at Sandia National Laboratory. All LAMMPS simulations were performed using a time step of 1 fs. For temperature and pressure regulation, the Nose–Hoover thermostat and barostat were applied during NVT and NPT ensembles, respectively [42,43]. Long-range electrostatic interactions were computed using the PPPM (particle–particle particle–mesh) method [44] with an accuracy of 1.0 × 10^−5^.

Following the preparation of amorphous models, crosslinking simulation was conducted to achieve different models with 0%, 10%, 30%, 50%, 70%, and 90% conversion levels. The conversion level is defined by following Equation (2):

(2) $$conversion\;level\left(\%\right)=\frac{number\;of\;crosslinked\;epoxide}{total\;number\;of\;epoxide}\times 100(\%)$$

**Figure 2:**
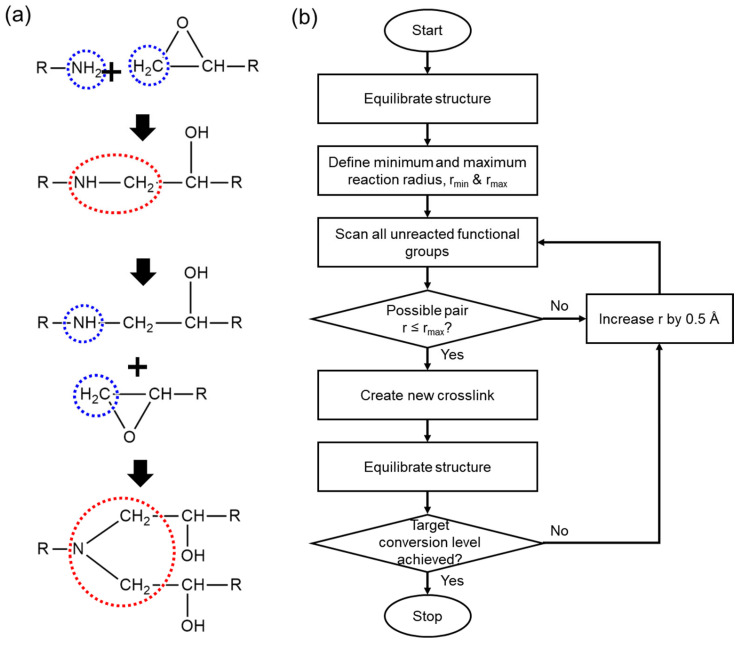
(**a**) Illustration of ring opening reaction during epoxy crosslinking, where blue and red dashed circles indicate reactive sites and the resulting bond formation, respectively. (**b**) flowchart of crosslinking simulation.

The crosslinking simulation was performed based on the crosslinking mechanism illustrated in Figure 2a. As described in Figure 2a, a ring-opening reaction, in which hydrogen atoms participate, occurs between the epoxide group of the base resin and the amine groups of the curing agents during the crosslinking process. The specific crosslinking simulation flowchart is demonstrated in Figure 2b. The simulation was designed based on the intermolecular curing mechanism, and we developed an in-house code to induce ring opening reactions depending on the intermolecular distances. In our approach, the bond connection occurs when the nitrogen atom of a curing agent comes within a defined range (r) from the carbon atom in the epoxide ring. This atom-specific criterion enables selective bond formation between chemically active sites. It is worth noting that previous studies have provided detailed descriptions of curing mechanicsm using activation energy-based models [45,46]. In contrast, our study employs a distance-based crosslinking algorithm, which is widely used in molecular dynamics simulations due to its computational efficiency and its ability to reliably capture the key structural features of crosslinked networks [47,48]. This approach allowed us to focus on investigating how subtle structural differences in curing agents affect network formation and thermomechanical behavior, without incurring excessive computational costs. As illustrated in Figure 2b, we established a range of minimum and maximum intermolecular distances, which are r_min_ (4 Å) and r_max_ (10 Å), respectively, to facilitate the formation of crosslinks within a defined distance. Consequently, we were able to obtain molecular models with certain degrees of curing and to investigate and measure how the properties are influenced by the crosslinking ratio.

Upon constructing initial models for each crosslinking ratio, we employed an annealing procedure to quickly and effectively optimize them for equilibrated structures. The temperature annealing procedure is described as follows: (a) The initial structure’s temperature was adjusted to 298.15 K with a slight contraction over a 100 ps period. (b) A gradual increment in temperature to 600 K was achieved through NVT simulation spanning 100 ps. (c) Sustaining the temperature at 600 K was maintained for an additional 100 ps via NVT simulation. (d) Subsequently, the temperature underwent a gradual reduction to 298.15 K through NVT simulation. (e) Repeat steps (b)–(d) three times. (f) The simulation model was brought to equilibrium through a 100 ps NVT MD simulation at 298.15 K and followed by a subsequent 500 ps.

After the optimization process was completed, 10 ns of NPT MD simulation was performed to obtain the equilibrated structures. To investigate the density of each crosslinking ratio model, we analyze the additional 5 ns of the NPT MD simulation for data collection. The stability of the thermodynamic properties of 3,3′-DDS and 4,4′-DDS systems during this phase, which are density, potential energy, and temperature, was verified to confirm equilibration as shown in Figure S1. Utilizing validated models obtained through density investigations, MD simulations according to temperature and strain changes were performed to derive thermal and mechanical properties, respectively. The temperature of the equilibrated model was elevated to 600 K for 3 ns and sustained for equilibration at higher temperatures during 5 ns. Subsequently, the simulation model’s density and volume changes were analyzed to obtain thermal properties, glass transition temperature (T_g_), and a coefficient of linear thermal expansion (CLTE) during the cooling procedure down to 200 K with cooling rate of 0.1 K/ps. The cooling rate of 0.1 K/ps used in this study is widely accepted in molecular dynamics simulations due to computational constraints and has been shown to be effective for capturing relative trends in thermal behavior [49,50,51,52]. T_g_ was determined by plotting the density values against temperature, identifying points where the slope exhibited a sharp change. The CLTE of glassy phase was determined by analyzing the slope of the volume change graph with respect to temperature prior to reaching the T_g_ point. Mechanical property, which is Young’s modulus, was investigated by subjecting the equilibrated model to tensile strain along three axes for 3 ns, reaching 30% strain. This corresponds to a strain rate of approximately 10^8^ s^−1^, which is commonly used in MD simulations due to timescale limitations [53,54,55,56]. The modulus was calculated from the initial linear region (0 to 3%) of the stress–strain curve, and values from the three directions were averaged to predict the system’s mechanical property.

## 3. Results and Discussion

### 3.1. Densities

**Figure 3:**
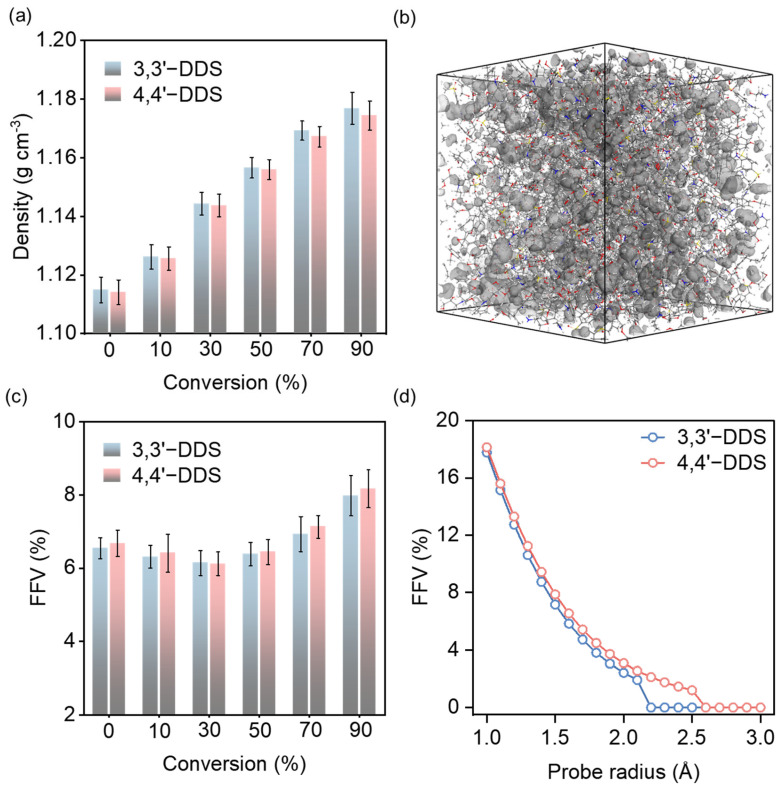
(**a**) Density analysis for simulation models cured with different curing agents with various conversion levels. (**b**) Connolly surface analysis method utilizing MD simulation, where gray regions represent the calculated free volume. (**c**) fractional free volume (FFV) at each conversion level. (**d**) FFV calculated as a function of probe radius.

The average density for each conversion level of the crosslinked epoxy models was investigated, as shown in Figure 3a. The graph shows that density increases with higher conversion levels. At 90% conversion, the densities of the 3,3′-DDS and 4,4′-DDS models are 1.1769 ± 0.0054 g cm^−3^ and 1.1744 ± 0.0049 g cm^−3^, respectively. Furthermore, the 3,3′-DDS model consistently has a slightly higher density than the 4,4′-DDS model at all conversion levels. As the conversion level increases, the development of the crosslinking network leads to a denser molecular structure, resulting in a gradual increase in density.

The observed effects of curing agents on density are consistent with the typical density variations between 3,3′-DDS and 4,4′-DDS reported in earlier studies [29,57,58]. Notably, this level of difference is comparable to those reported in previous simulation studies on isomeric epoxy systems, where small structural variations resulted in similarly subtle density differences [20,28]. Experimental studies have reported that the density of 3,3′-DDS systems is approximately 1.273 g cm^−3^, whereas that of 4,4′-DDS systems is around 1.253 g cm^−3^, indicating that 3,3′-DDS consistently exhibits a higher density trend [59]. Compared to these experimental values, our MD simulations yield density values that are approximately 8% lower, which is consistent with previous findings that molecular dynamics models tend to underestimate density relative to experimental measurements. This discrepancy is primarily attributed to the inherent limitations of classical force fields and system size effects in MD simulations, as reported in previous computational studies [60,61]. Importantly, the relative density trend remains consistent, supporting the validity of our model in capturing structural differences between the curing agents. Furthermore, Amariutei et al. [16] describe how the differing configurational structures of the two curing agents affect density by considering each structure’s packing efficiency. As a result of comparing the density of the two curing agents’ systems, the packing efficiency of the curing agents can be estimated. Because of its conformational diversity, the meta structure of 3,3′-DDS packs more efficiently than the para structure [16,17,20,62].

To complement this observation, fractional free volume (FFV) was evaluated using Connolly surface analysis with a probe radius of 1.4 Å, which corresponds to the typical molecular radius of water. The visualization of this method is shown in Figure 3b. As shown in Figure 3c, FFV decreases from 0% to 30% conversion as scattered molecules form crosslinks, then increases beyond 30% as a more rigid network is developed. At all conversion levels, the 3,3′-DDS system exhibits lower FFV than the 4,4′-DDS system, supporting the interpretation that the meta-substituted structure results in more efficient packing. Further analysis at 90% conversion using varying probe radii (1–3 Å) revealed that the 4,4′-DDS system possesses not only greater total fractional free volume (FFV) but also larger voids accessible to bigger probes, as shown in Figure 3d. This indicates that 4,4′-DDS networks contain more interconnected or expansive free volume pockets, consistent with its relatively lower packing efficiency. These findings further support the interpretation that 3,3′-DDS forms a more compact molecular structure, characterized by smaller and more confined free volume domains.

This enhanced molecular adaptability promotes denser network formation with the base resin, leading to improved packing efficiency. This structural behavior aligns with previous findings on how molecular conformation influences packing efficiency. Discrepancies between experimental and simulated density values are frequently caused by the simulation scale, which usually results in lower simulated densities. Nonetheless, the consistency of the density trends for 3,3′-DDS and 4,4′-DDS with experimental values confirms the well-established character of the simulation model used in this study [16,17,20].

### 3.2. Thermal Properties

**Figure 4:**
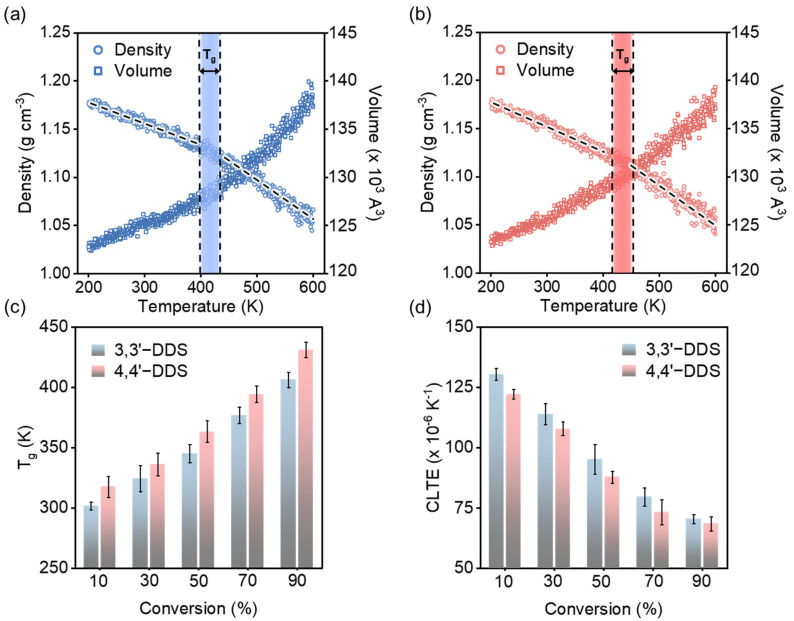
T_g_ and CTE were analyzed for DGEBF cured with (**a**) 3,3′-DDS and (**b**) 4,4′-DDS. (**c**) T_g_ and (**d**) CLTE values from the simulation model for each conversion level.

Thermal characteristics are important descriptors of a polymer system’s thermal stability and resilience. The determination of the glass transition temperature (T_g_) is particularly crucial, as it provides information on the system’s ability to endure and respond to temperature changes. The molecular structure, component mobility, and crosslink density within the material all have a substantial impact on thermal characteristics in these systems. The T_g_ can be determined through simulation techniques involving the plotting of density or volume against temperature. By identifying the point where the slope exhibits a sharp change during cooling from 600 K to 200 K, the transition from a rubbery to a glassy state, indicating T_g_, can be discerned. Additionally, we computed the coefficient of linear thermal expansion (CLTE) in the glassy phase by evaluating the rate of volume change concerning temperature. Equation (3) outlines the procedure for calculating CLTE based on the slope of volume changes:

(3) $$CLTE=\frac{1}{3V_{0}}(\frac{dV}{dT})$$

where $V_{0},\;dV$ and dT are initial volume at room temperature, 298.15K, change in volume, and change in temperature, respectively. Figure 4a,b show the temperature-induced density and volume variations for DGEBF with 3,3′-DDS and 4,4′-DDS, respectively, both cured at 90% conversion.

Figure 4c illustrates the derived T_g_ values for models utilizing various curing agents and conversion levels. T_g_ values for the 3,3′-DDS and 4,4′-DDS models increase with the conversion level, reaching 406.36 ± 6.39 K and 431.22 ± 5.28 K at the 90% conversion level, respectively. These values are consistent with experimentally obtained results [59,63,64]. Across all conversion levels, the 4,4′-DDS model consistently outperforms the 3,3′-DDS model by over 10 °C. This discrepancy is attributed to structural differences in the curing agents, specifically the position of the functional group connected to the phenylene ring. The structural difference between the meta and para configurations of 3,3′-DDS and 4,4′-DDS influences the free volume, affecting the packing efficiency of the crosslinked network [16,17,20,62,65].

CLTE, as a measure of how much a material changes with temperature, becomes especially significant in comparing high-performance epoxy systems due to epoxy’s brittle nature, which can result in damage if volume changes occur abruptly [66]. Figure 4d presents CLTE values for each conversion level and curing agent. The CLTE of both 3,3′-DDS and 4,4′-DDS models decreases from 130.47 ± 2.48 × 10^−6^ K^−1^ to 70.46 ± 1.88 × 10^−6^ K^−1^ and 122.27 ± 1.94 × 10^−6^ K^−1^ to 68.53 ± 2.94 × 10^−6^ K^−1^, respectively, as the conversion level increases from 10% to 90%. At lower conversion levels, the reduced molecular crosslink density enables easier expansion with rising temperature, leading to higher CLTE values. Conversely, at higher conversion levels, the increased crosslink density resulting from polymer chains restricts volume expansion even as temperature increases. When comparing the T_g_ values of the 3,3′-DDS and 4,4′-DDS models, the 4,4′-DDS model exhibits lower CLTE values than the 3,3′-DDS system because its higher T_g_ value indicates less temperature transition.

**Figure 5:**
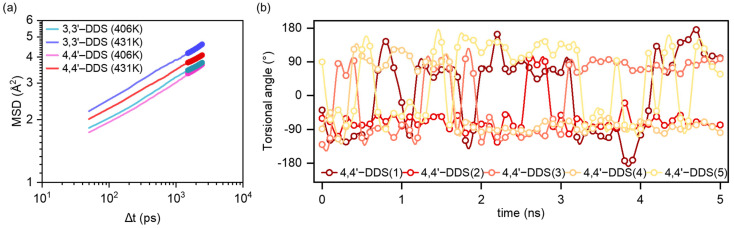
(**a**) MSD profiles of 3,3′-DDS and 4,4′-DDS systems at 406.36 K and 431.22 K. (**b**) Representative torsional angle analysis of five cases in 4,4′-DDS during a 5 ns simulation at 406.36 K.

Since the glass transition is closely related to the onset of molecular motion in the polymer network, we investigated whether the two systems exhibit different mobility at both T_g_ values. To evaluate this, we analyzed the mean squared displacement (MSD), which quantifies the time-averaged displacement of atoms and is commonly used to assess molecular motion in condensed-phase systems. The MSD and diffusion coefficient were calculated using the following equations:

(4) $$\mathrm{MSD}\left(t\right)=\langle {\left|r\left(t\right)-r\left(0\right)\right|}^{2}\rangle$$

(5) $$D=\frac{1}{6}\frac{d}{dt}MSD(t)$$

where r(t) and r(0) represents the atomic position at time t and at the initial time, respectively; the angle brackets indicate an ensemble average. Equation (4) describes the spatial displacement over times, while Equation (5) yields the diffusion coefficient D, which represents long-range mobility in the system. As shown in Figure 5a and Table S1, at 406.36 K, which corresponds to the T_g_ of the 3,3′-DDS system, its MSD is already higher than that of the 4,4′-DDS system. At 406.36K, the diffusion coefficients for 3,3′-DDS and 4,4′-DDS systems are 5.27 and 5.41 × 10^−9^ cm^2^ s^−1^, respectively. This difference becomes more distinct at 431.22 K, the T_g_ of the 4,4′-DDS system, where the 3,3′-DDS model shows greater displacement. At 431.22 K, the diffusion coefficient of 3,3′-DDS increases significantly to 7.98 × 10^−9^ cm^2^ s^−1^, while 4,4′-DDS remains at 5.59 × 10^−9^ cm^2^ s^−1^. These results indicate that, despite its denser and more tightly packed structure, the 3,3′-DDS system reaches the onset of molecular motion at a lower temperature.

Tu et al. [62] suggested that the flipping motion of para-substituted benzene rings can dissipate local energy and contribute to an increased T_g_. Motivated by this hypothesis, we tracked the torsional angle fluctuations of the benzene rings in our simulation models at 406.36 K, which corresponds to the T_g_ of the 3,3′-DDS system, for 5 ns to determine whether such localized motion occurs. The results of this analysis are presented in Figure S2. Among the tracked data, the representative behavior of five cases in the 4,4′-DDS system is shown in Figure 5b. As shown in Figure 5b, the 4,4′-DDS system exhibits active ring flipping, whereas the 3,3′-DDS system shows minimal torsional fluctuation. According to the results shown in Figure S2, no ring flip events were observed in the 3,3′-DDS system, while a total 52 ring flips events were identified in the 4,4′-DDS system. The linear para-substituted 4,4′-DDS facilitates flip rotation, while the meta-substituted 3,3′-DDS restricts such motion due to its bent geometry.

### 3.3. Mechanical Properties

**Figure 6:**
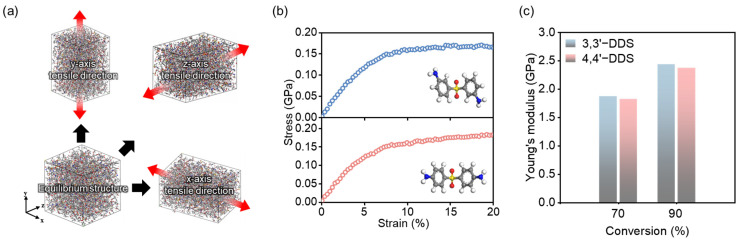
(**a**) Uniaxial tensile deformation was applied along the x-, y-, and z-directions. (**b**) Stress–strain curves of 3,3′-DDS (top) and 4,4′-DDS (bottom) systems at 90% conversion. (**c**) Young’s modulus of each system at 70% and 90% conversion, calculated from the initial linear region.

Figure 6a illustrates the method of applying deformation to the model in each axis direction to measure stress, which is utilized to evaluate the mechanical properties of the simulation model. It is noted that below the theoretical gel point (~57.7% cure) [67], the system exhibits liquid-like behavior; therefore, the Young’s modulus was measured at a high degree of conversion.

To ensure elastic behavior is captured, the modulus was calculated as the slope of the stress–strain curve within the initial linear region. Figure 6b displays the stress–strain responses for each curing agent at 90% conversion from which the Young’s modulus was calculated using the initial linear region. Figure 6c shows that the modulus increases with conversion level in both systems. At a curing ratio of 70%, the modulus of the 3,3′-DDS system is 1.87 GPa, while that of the 4,4′-DDS system is 1.83 GPa. At 90% conversion, the modulus values further increase to 2.44 GPa and 2.38 GPa, respectively, confirming that the 3,3′-DDS system consistently exhibits a slightly higher Young’s modulus. This trend remains consistent across all curing ratios. As discussed in Section 3.1, the 3,3′-DDS system exhibits higher density and more efficient packing, which contributes to its increased modulus. Consistent with previous studies, the 3,3′-DDS system demonstrates higher modulus values attributed to the enhanced conformational diversity and packing efficiency of 3,3′-DDS [20,68]. The increased packing efficiency implies closer and stronger molecular connections, resulting in the entire system being more resistant to deformation forces. Additionally, the CED was calculated through an analysis of non-bonded energy within the system. CED can be determined using Equations (4) and (5):

(6) $$E_{coh}=-E_{\mathrm{non-bonded}}=-\left(E_{vdW}+E_{Q}\right)$$

(7) $$CED=\frac{E_{coh}}{volume\;of\;the\;system}$$

where $E_{coh},\;E_{non-bonded,\;}\;E_{vdW}$ and $E_{Q}$ are cohesive, non-bonded, van der Waals, and electrostatic energy, respectively. As evident from the formula, CED indicates the density of non-bonded energy and serves as an indicator of cohesive strength between molecules [8,69]. Consequently, polymers with higher CED values tend to exhibit better mechanical properties due to enhanced overall molecular interaction [68,70]. The 3,3′-DDS model has a larger CED value than the 4,4′-DDS model, with values of 457.95 J cm^−3^ and 397.39 J cm^−3^, respectively. When comparing the modulus of 3,3′-DDS to 4,4′-DDS, the higher CED of 3,3′-DDS corresponds to enhanced mechanical characteristics, consistent with previous research findings [20,68].

## 4. Conclusions

This paper investigates how structural differences between two aromatic amine curing agents, 3,3′-DDS and 4,4′-DDS, affect the thermomechanical behavior of DGEBF-based epoxy systems. Based on molecular dynamics simulations and structural analysis, the main conclusions are as follows:

(1) As the conversion level increases from 0% to 90%, the epoxy network becomes denser, resulting in increased density, glass transition temperature (T_g_), and Young’s modulus. Conversely, the coefficient of linear thermal expansion (CLTE) decreases with higher conversion.
(2) Compared to the 4,4′-DDS system, the 3,3′-DDS system consistently exhibits higher density and Young’s modulus across all conversion levels. Meanwhile, the 4,4′-DDS system exhibits a higher T_g_ and a lower CLTE, highlighting the influence of crosslinking agents on the bulk properties.
(3) Mean squared displacement (MSD) analysis revealed that the 3,3′-DDS system reaches the onset of molecular motion at a lower temperature compared to the 4,4′-DDS system, despite its higher density and packing efficiency. This dynamic behavior accounts for the lower T_g_ of the 3,3′-DDS system and further implies an influence on its thermal relaxation characteristics.
(4) The higher Young’s modulus observed in the 3,3′-DDS system is attributed to its denser network structure and greater packing efficiency, which result in enhanced intermolecular cohesion. This is supported by higher CED values, indicating stronger non-bonded interactions that contribute to the increased stiffness of the network.

This study provides molecular-level insight into how subtle geometric differences in curing agent structure affect network packing, mobility, and macroscopic performance, thereby guiding the development of high-performance epoxy systems.

## Data Availability

Data are contained within the article and Supplementary Materials.

## Supplementary Materials

The following supporting information can be downloaded at: https://www.mdpi.com/article/10.3390/polym17121694/s1, Table S1. Diffusion coefficients (D) of the epoxy systems at two temperatures corresponding to the T_g_ of each curing agent (406.36 K for 3,3′-DDS and 431.22 K for 4,4′-DDS). Figure S1. Time evolution of thermodynamic properties during the final 5 ns data collection simulation at 298 K. (a–c) show density, potential energy, and temperature profiles for the 3,3′-DDS system; (d–f) show the corresponding results for the 4,4′-DDS system. Figure S2. Torsional angle analysis of benzene rings in each curing agent during a 5 ns simulation at 406.36 K, where (a) corresponds to 3,3′-DDS and (b) to 4,4′-DDS.
