# Supplementary material for: Molecular Level Understanding of Amine Structural Variations on Diaminodiphenyl Sulfone to Thermomechanical Characteristics in Bifunctional Epoxy Resin: Molecular Dynamics Simulation Approach
Source: Polymers (Basel). 2025 Jun 18;17(12):1694. doi: 10.3390/polym17121694 (PMC12196684; doi:10.3390/polym17121694)
Supplement: Supplementary file 1 [file polymers-17-01694-s001.zip › polymers-3617433-supplementary.pdf]

# Supporting Information

## Molecular Level Understanding of Amine Structural Variations on Diaminodiphenyl Sulfone to Thermomechanical Characteristics in Bifunctional Epoxy Resin: Molecular Dynamics Simulation Approach

Hei Je Jeong[1,†], Sung Hyun Kwon[2,3,†], Jihoon Lim[2], Woong Kwon[4], Gun Hwan Park[4], Eunhye Lee[5], Jong Sung Won[5], Man Young Lee[5], Euigyung Jeong[4,*] and Seung Geol Lee[1,*]

[1] Department of Materials Science and Engineering, Ulsan National Institute of Science and Technology (UNIST), Ulsan 44919, Republic of Korea

[2] School of Chemical Engineering, Pusan National University, Busan 46241, Republic of Korea

[3] Department of Organic Material Science and Engineering, Pusan National University, Busan 46241, Republic of Korea

[4] Department of Textile System Engineering, Kyungpook National University, Daegu 41566, Republic of Korea

[5] Defense Material and Energy Development Center, Agency for Defense Development, Yuseong P.O. Box 35, Daejeon 34060, Republic of Korea

*Corresponding authors:
wolfpack@knu.ac.kr (E.J.)
seunggeol.lee@pusan.ac.kr (S.G.L.)
[†] These authors contributed equally to this study.

**Table S1.** Diffusion coefficients (D) of the epoxy systems at two temperatures corresponding to the $T_g$ of each curing agent (406.36 K for 3,3′−DDS and 431.22 K for 4,4′−DDS).

| D (x $10^{-9}$ $cm^2$ $s^{-1}$) | 3,3'−DDS | 4,4'−DDS |
|---|---|---|
| at 406.36 K | 5.27 | 5.41 |
| at 431.22 K | 7.98 | 5.59 |

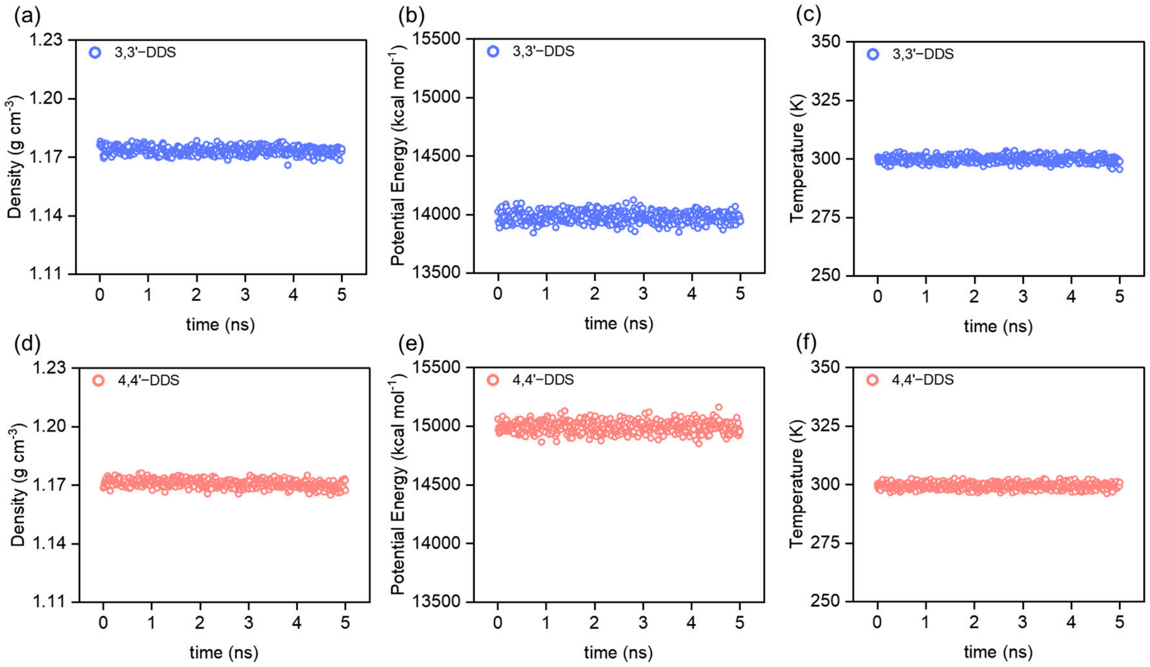


**Figure S1.** Time evolution of thermodynamic properties during the final 5 ns data collection simulation at 298 K. (a–c) show density, potential energy, and temperature profiles for the 3,3′−DDS system; (d–f) show the corresponding results for the 4,4′−DDS system.

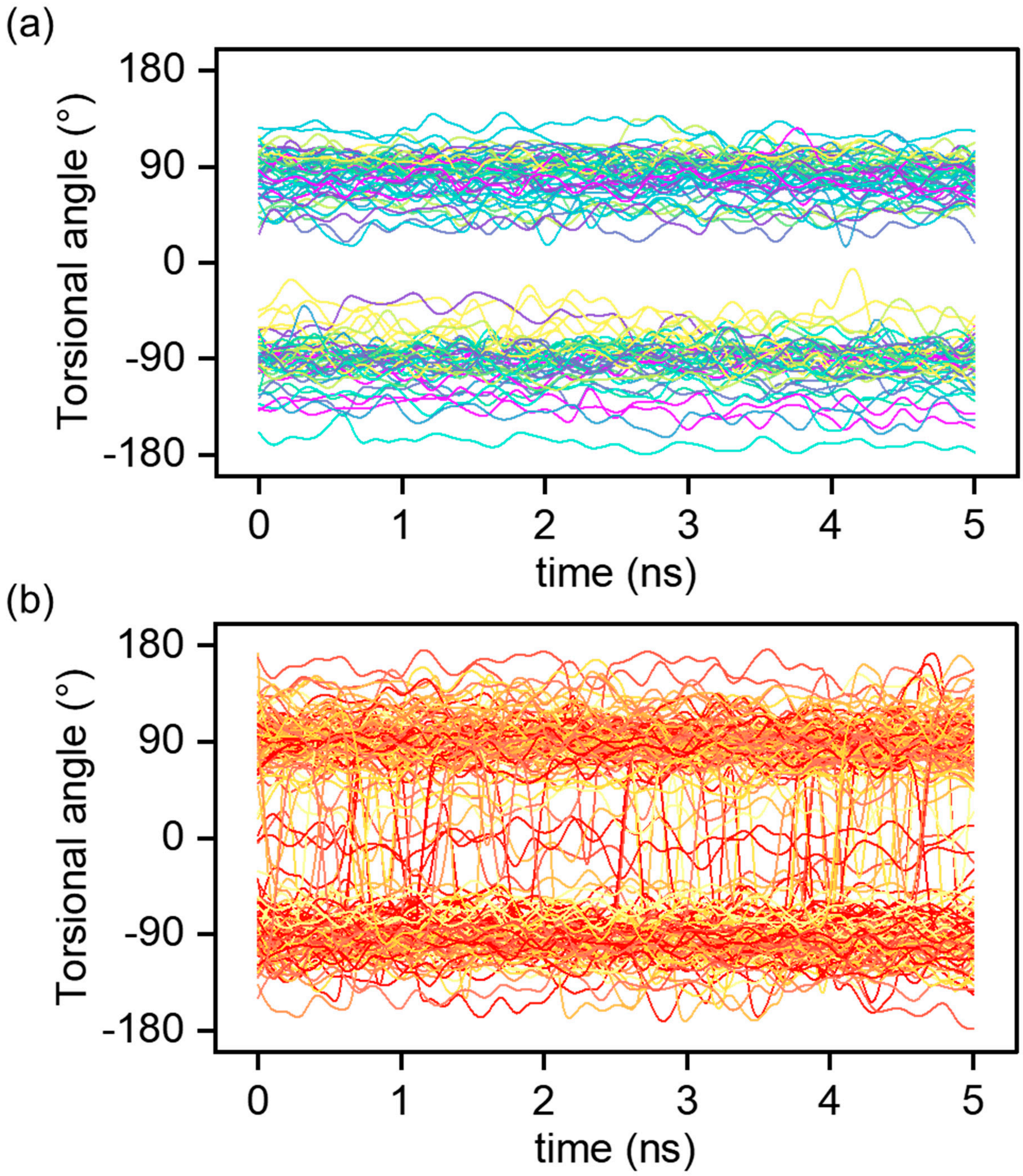


**Figure S2.** Torsional angle analysis of benzene rings in each curing agent during a 5 ns simulation at 406.36 K, where (a) corresponds to 3,3′−DDS and (b) to 4,4′−DDS. This figure presents the full torsional trajectories and highlights the difference in dynamic behavior between the two isomers.
